# Decoding Imagined and Spoken Phrases From Non-invasive Neural (MEG) Signals

**DOI:** 10.3389/fnins.2020.00290

**Published:** 2020-04-07

**Authors:** Debadatta Dash, Paul Ferrari, Jun Wang

**Affiliations:** ^1^Department of Electrical and Computer Engineering, University of Texas at Austin, Austin, TX, United States; ^2^Department of Neurology, Dell Medical School, University of Texas at Austin, Austin, TX, United States; ^3^MEG Lab, Dell Children's Medical Center, Austin, TX, United States; ^4^Department of Psychology, University of Texas at Austin, Austin, TX, United States; ^5^Department of Communication Sciences and Disorders, University of Texas at Austin, Austin, TX, United States

**Keywords:** MEG, speech, brain-computer interface, wavelet, convolutional neural network, neural technology

## Abstract

Speech production is a hierarchical mechanism involving the synchronization of the brain and the oral articulators, where the intention of linguistic concepts is transformed into meaningful sounds. Individuals with locked-in syndrome (fully paralyzed but aware) lose their motor ability completely including articulation and even eyeball movement. The neural pathway may be the only option to resume a certain level of communication for these patients. Current brain-computer interfaces (BCIs) use patients' visual and attentional correlates to build communication, resulting in a slow communication rate (a few words per minute). Direct decoding of imagined speech from the neural signals (and then driving a speech synthesizer) has the potential for a higher communication rate. In this study, we investigated the decoding of five imagined and spoken phrases from single-trial, non-invasive magnetoencephalography (MEG) signals collected from eight adult subjects. Two machine learning algorithms were used. One was an artificial neural network (ANN) with statistical features as the baseline approach. The other was convolutional neural networks (CNNs) applied on the spatial, spectral and temporal features extracted from the MEG signals. Experimental results indicated the possibility to decode imagined and spoken phrases directly from neuromagnetic signals. CNNs were found to be highly effective with an average decoding accuracy of up to 93% for the imagined and 96% for the spoken phrases.

## 1. Introduction

Speech is an essential attribute of humans, with the execution of verbal communication being underpinned by a very complex—yet poorly understood—relationship between neural processing and articulation. Speech centers of the brain including primary motor regions in synchrony with the articulators guide the mechanism of speech production where thoughts are transformed into meaningful words in the form of acoustics (Levelt, 1999; Ackermann, 2008). Brain damage or late-stage neurodegenerative diseases such as amyotrophic lateral sclerosis (ALS) leads to a state called locked-in syndrome, where patients are cognitively intact but motorically “locked-in” (Smith and Delargy, 2005; Kiernan et al., 2011). There is a population incidence of about 0.7/10, 000 for the locked-in syndrome (Kohnen et al., 2013). Communication assistance is critical for these patients to resume a meaningful life. Since the whole body, including the articulators, fingers, and eyes are paralyzed, a motoric bypass directly utilizing brain activity might be the only option to reestablish their communication. Electroencephalography (EEG) is the standard modality from which cortical potentials, P300, or sensory-motor rhythm (SMR) oscillations are used for assessing the brain dynamics in brain-computer interfaces (BCIs) (Brumberg et al., 2010). EEG is a reasonable choice for brain-based communication for patients with debilitating neurodegenerative diseases, primarily because of its non-invasiveness, low cost, and satisfactory temporal resolution (Birbaumer, 2006). However, the major disadvantage of current EEG-BCIs is the slow word synthesis rate which is about a few words (< 10) per minute (Birbaumer, 2006). This is mostly due to the passive letter selection paradigm of the EEG-BCI designs where subjects are required to select control characters randomly displayed on a screen prompted with visual or attention correlates. A direct neural speech decoding approach may improve efficacy by providing a faster communication rate than the current BCIs. In this framework, once the imagined- or intended-speech is generated internally, these signals are then decoded to text or speech parameters, and then a text-to-speech synthesizer (Cao et al., 2017) or a vocoder (Akbari et al., 2019) can be used to construct speech immediately.

While a number of speech decoding studies have been conducted using EEG recently such as for classification of imagined syllables (D'Zmura et al., 2009; Brigham and Vijaya Kumar, 2010; Deng et al., 2010), isolated phonemes (Chi and John, 2011; Leuthardt et al., 2011; Zhao and Rudzicz, 2015; Yoshimura et al., 2016), alphabets (Wang et al., 2018), or words (Porbadnigk et al., 2009; Nguyen et al., 2017; Rezazadeh Sereshkeh et al., 2017), the decoding performances have been intermediate, e.g., 63.45% for a binary (yes/no) classification (Rezazadeh Sereshkeh et al., 2017) or 35.68% for five vowel classification (Cooney et al., 2019a). There are inherent disadvantages in using EEG that may have contributed to the difficulty in attaining high decoding performance. For example, EEG recorded signals are distorted by neural tissue boundaries, skull, and scalp. Additionally, EEG is reference-based and has a relatively lower spatial resolution. Functional magnetic resonance imaging (fMRI), which has a high spatial resolution, has also been used for speech decoding but only during speech perception, speech categorization, and speaker recognition (Formisano et al., 2008). Although these studies are important for understanding the neural speech perception mechanism, decoding speech perception is not adequate to drive a speech-BCI for intended/imagined speech production. Furthermore, fMRI has a low temporal resolution (Dash et al., 2018a,b) and hence is not suitable for decoding speech production. Very recently, Electrocorticography (ECoG) has shown great potential for direct neural speech decoding of spoken, isolated phonemes (Ramsey et al., 2018), words (Kellis et al., 2010; Martin et al., 2016), and even of continuous speech (open set phrases) (Herff et al., 2015). Direct synthesis of speech from neural signals has also been shown to be possible with ECoG (Angrick et al., 2019; Anumanchipalli et al., 2019). However, ECoG requires a craniotomy and surgical placement of electrodes into the brain, which presents a challenge for establishing bio-compatibility between the device and the brain for long-term use. In addition, with ECoG, only a part of the brain (usually speech centers) is utilized as it is extremely impractical, if not impossible, to implant electrodes across the whole brain. Thus, a non-invasive, high temporal resolution, whole-head neuroimaging modality holds the potential for the development of future BCIs with a faster communication rate.

The current focus of neural decoding has been on either overt speech (Dash et al., 2018d; Livezey et al., 2019) or imagined (covert) speech, which corresponds to imagining speech pronunciation in the absence of articulatory and acoustic output (D'Zmura et al., 2009; Yoshimura et al., 2016; Rezazadeh Sereshkeh et al., 2017; Cooney et al., 2018). Considering the behavioral difficulty in investigating imagined speech, it is understandable that the majority of the speech-BCI research is dominated by overt speech decoding studies. Overt speech performance can be verified with the produced acoustic output whereas the verification of imagined speech production is ambiguous, indefinite, and subjective. In fact, the current decoding studies involving open-set brain to text (Herff et al., 2015; Moses et al., 2019) or brain to speech (Angrick et al., 2019; Anumanchipalli et al., 2019) decoding are on overt speech. Current neural decoding of imagined or intended speech is still limited to closed-set classifications (Guenther et al., 2009; Brumberg et al., 2011; Ikeda et al., 2014; Nguyen et al., 2017; Cooney et al., 2018). For instance, using EEG, researchers have successfully performed imagined speech decoding by classifying various short speech units, e.g., two syllables (D'Zmura et al., 2009), five phonemes (Chi and John, 2011), two vowels (Iqbal et al., 2015; Yoshimura et al., 2016), seven phonemes (Zhao and Rudzicz, 2015), and even words (Porbadnigk et al., 2009; Nguyen et al., 2017; Rezazadeh Sereshkeh et al., 2017; Hashim et al., 2018). Studies using ECoG have also shown the possibility of decoding imagined speech (Ikeda et al., 2014; Martin et al., 2016). However, there is still room for improvement in the accuracies obtained in all of these imagined speech decoding studies. There is some evidence from fMRI that imagined speech produces lower levels of brain activity compared to overt speech (Palmer et al., 2001; Shuster and Lemieux, 2005), which may explain the lower decoding performance of the former in literature. In short, there is a need for improved performance of decoding imagined speech.

In this study, we performed decoding of both imagined and overt speech production. Instead of using isolated phonemes or syllables, we collected neural data during the imagination and production of phrases (e.g., how are you?), with the eventual goal of open-vocabulary decoding (decoding phonemes within phrases) for naturalistic communication (Iljina et al., 2017). Here, we classified whole phrases, as a starting point. The neurolinguistics underpinnings supporting phrase-level covert or overt articulation is widely studied topic, but has not yet been explored in a decoding experiment (Memarian et al., 2012). Furthermore, acknowledging the difficulty in verifying the behavioral compliance of imagined speech production (Cooney et al., 2018), in contrast to the data acquisition paradigm of current literature for separately collecting data for overt and imagined speech, we collected the neural signals corresponding to imagined and overt speech consecutively, within the same trial, where the timing of this paradigm constrained the subjects to imagining/preparing the same phrase he/she is expected to articulate for the trial.

We used magnetoencephalography (MEG) to record the neuromagnetic signals corresponding to speech imagination and production. Magnetoencephalography (MEG) is a non-invasive, whole-head neuroimaging modality that uses highly sensitive magnetometers and gradiometers to record the magnetic fields associated with intracellular post-synaptic neuronal currents in the brain (Cohen and Cuffin, 1983). Unlike the electric signals measured with EEG and ECoG, the magnetic field signals measured by MEG pass through the dura, skull, and scalp relatively undistorted, and thus provide a more accurate representation of the underlying brain activities. MEG has a higher spatial resolution than EEG while maintaining a very high temporal resolution (1 ms or even lower). These unique benefits make MEG a great fit for the investigation of speech decoding. Recent MEG based speech studies suggest the efficacy of MEG in capturing the fast temporal dynamics of the speech signal (Simos et al., 1998; Memarian et al., 2012; Wang et al., 2017; Dash et al., 2018d, 2019b), and provide further evidence in support of the use of neuromagnetic signals to be used in speech decoding. Although current MEG machines are non-portable and costly, a recent study on wearable MEG (Boto et al., 2018) showed the potential of building next-generation, portable MEG devices. Further, unlike SQUID based measurement system, it uses optically pumped magnetometers (OPMs) which can reduce the cost dramatically. These recent advances in technology hold great promise for suitable MEG mediated speech-BCI applications in the near future.

Three decoding approaches were tested in this experiment. First, we used an artificial neural network (ANN) trained on the root mean square (RMS) features of the neural signals. We considered this approach as our baseline, which was used in our pilot studies (Dash et al., 2018c,d). Considering the difficulty in collecting lots of neural signal data, previously, researchers have employed simpler decoders for classification equivalent to our baseline approach such as matched filter on Hilbert envelope features (D'Zmura et al., 2009), Bayesian classifier based on multi-class linear discriminant analysis (LDA) on Hilbert envelope features (Deng et al., 2010), or spectral features (Chi and John, 2011), nearest neighbor classification on the features extracted with Euclidian distance of the coefficients from autoregressive models (Brigham and Vijaya Kumar, 2010), support vector machine on statistical features (Zhao and Rudzicz, 2015), and Euclidian distance feature (Martin et al., 2016), relevance vector machine on Riemannian manifold features (Nguyen et al., 2017), hidden Markov model on temporal neural signals (Porbadnigk et al., 2009), artificial neural network on Wavelet-transform based statistical features (root mean square and standard deviation) (Rezazadeh Sereshkeh et al., 2017), etc. Among all, ANN with wavelet-transform based statistical features (Rezazadeh Sereshkeh et al., 2017) has shown comparatively better decoding performance which inspired our pilot studies and baseline of this study for exploring with statistical features and using ANN as the decoder. Second, we employed convolutional neural networks (CNNs) trained on spectral-temporal features in terms of scalograms of the neuromagnetic signals. Third, to further utilize the neural information, we added spatial dimension on top of the second approach. In other words, CNNs were trained using spatial-spectral-temporal features in the third approach. CNNs have recently shown great potential in a wide variety of application in computer vision, and acoustic speech decoding, which outperform ANNs. CNNs are inspired by visual cortex architecture of the brain where the cortical neurons work on a restricted area of the visual domain (called receptive field) by partial overlapping with each other to cover the whole visual space. CNNs are functionally very similar to the traditional neural networks as it operates as a variation of multilayer perceptions, but are modeled to require minimal processing (Cireşan et al., 2011). In Roy et al. (2019), it is reported that a total of 40% studies amongst all research involving deep learning applications to EEG have used CNNs, but none of them were for speech decoding. Nevertheless, the efficacy of CNN for neural data analysis can be translated for neural speech decoding which we experimented within this study. Moreover, a few recent studies have shown the efficacy of using CNN to analyze MEG (Hasasneh et al., 2018; Dash et al., 2019a; Huang and Yu, 2019) or EEG data (Cooney et al., 2019a,b), which further strengthens our motivation for this approach. To our knowledge, this is the first study using CNNs to explore neural speech decoding with MEG.

## 2. Dataset and Feature Extraction

### 2.1. Data Collection

Eight right-handed subjects (five males and three females) with a mean age of 41 years (standard deviation = 14 years) participated in the data collection. The subjects had normal or corrected to normal vision. No speech/language/hearing or cognitive history was reported from the subjects. All the subjects were English speakers. Written consent was obtained from each subject prior to the experiment. This study has been approved by the local ethics committees at the University of Texas at Dallas, the University of Texas at Austin, Dell Children's Medical Center (Austin, TX), and Cook Children's Hospital (Fort Worth, TX).

**Figure 1:**
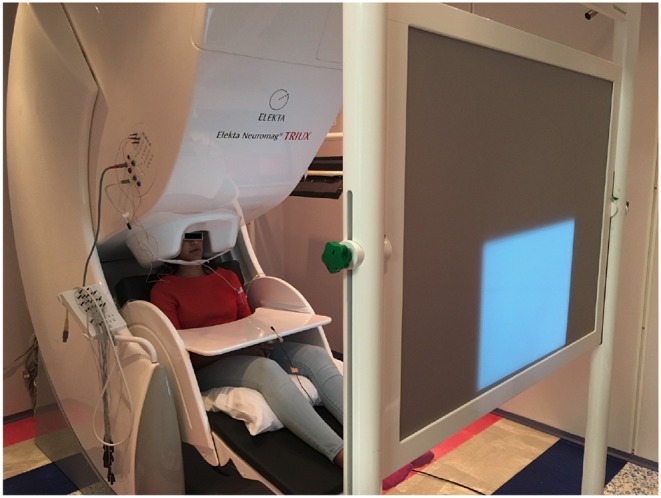
A MEG unit. The picture represents the Neuromag Eleckta Ltd. MEG machine situated within a magnetically shielded room at the Cook Children's Hospital, Texas. A subject is seated comfortably in the unit. The projected display is for showing the stimuli (text) in a pseudo-randomized order.

The data acquisition was performed at two places, one at the MEG Lab, Cook Children's Hospital where the data were collected from four subjects. The data for the other four subjects were collected at the MEG lab, Dell Children's Medical Center. The two hospitals have identical Elekta Neuromag Triux MEG devices as shown in Figure 1, which were used to record the brain activity signals. The machine consists of 306 channels with 204 planar gradiometers and 102 magnetometer sensors. It is housed within a magnetically shielded room (MSR) to discard any unwanted environmental magnetic field interferences. Prior to recording, the coordinate system based on three fiducial points (the left and right pre-auricular points and one at the nasion) was created for the subjects. For coregistration of the subjects within the MEG system, five head-position-coils were fixed to their head and digitalized using a Polhemus Fastrak, and then localized in the MEG at the start of each experimental run. The brain activity signals were acquired via MEG with 4 kHz sampling frequency which were then band-pass filtered and resampled to 1 kHz. Eye-blinking artifacts were collected through electrooculography (EOG) by two integrated sensors placed at the upper and lower aspect of the outer-canthi. The cardiac signal was recorded by two bipolar integrated electrocardiograph (ECG) sensors placed on top of the collarbone areas. Acoustic output during the speech production stage was recorded through a standard built-in microphone connected to a transducer placed outside the MSR. To record the jaw movement, a custom-made air bladder was used which was connected to an air pressure sensor. By recording the depression in that bladder jaw motion data during articulation was acquired. Both speech and jaw movement analog signals were then digitized by feeding into the MEG ADC in real-time as separate channels. All the sensors were checked for noise and calibrated prior to data collection. Subjects sat upright in the MEG with their hands resting on a platform in front of them. In order to reduce head movements, subjects underwent a few minutes of adjustments and training about slouching. Visual stimuli were generated by a computer running the STIM2 software (Compumedics, Ltd.), and presented via a DLP projector situated at 90 cm from the subjects'.

### 2.2. Experimental Protocol

**Figure 2:**
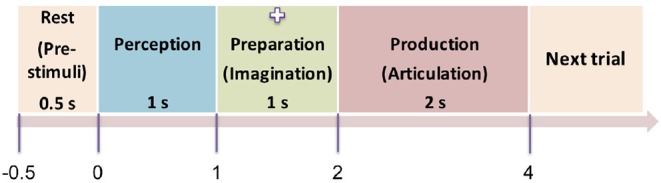
Protocol of the time-locked experiment. The four time-locked stages of the experiment (*rest* → *perception* → *preparation* → *articulation*) are shown here. The numbers represent the initial onset time of the stages. Next trial starts with a non-movement baseline of 1 s. The screen was blank in dark background during the pre-stimuli stage. A stimulus (text) was displayed on the screen during the perception segment, then replaced by a cross sign during the preparation stage. The cross disappeared (blank display) again during the production stage.

The experiment was designed as a delayed overt reading task, with four segments within each trial: pre-stimulus (rest), perception, preparation (imagination), and production (articulation) as shown in Figure 2. The pre-stimuli segment was designated as a period of 0.5 s prior to the stimulus onset. The perception segment was initiated by a single stimulus (phrase) being displayed on the screen for the subjects to covertly read. The stimulus was on the screen for 1 s after which it was replaced by a fixation cross (+). The duration of the fixation was 1 s which corresponded to the imagination (preparation) segment. For this segment, the subjects were previously instructed to think or imagine, and be prepared to speak. The removal of the fixation cross prompted the subjects to overtly articulate the previously viewed phrase at their natural speaking rate (production). The average time for production/articulation segment was 2 s (for one subject it was 1.5 s; for other two subjects it was 2 s, and for the rest of the 5 subjects it was 2.5 s) based on the natural speaking rate of the subjects. There was a 1−1.5 s of non-movement baseline prior to the next stimulus trial. This 4-stage procedure was repeated for 100 trials for each of the 5 stimuli. Five commonly used English phrases were used as stimuli, selected from the phrase lists that are used in alternative augmented communication (AAC) devices. They are: *phrase 1: Do you understand me, phrase 2: That's perfect, phrase 3: How are you, phrase 4: Good-bye, and phrase 5: I need help*. The presentation order of the stimuli was pseudo-randomized to avoid response suppression to repeated exposure (Grill-Spector et al., 2006; Cheyne and Ferrari, 2013). Subjects were trained on some sample stimuli before the experiment to ensure compliance. The whole experiment lasted approximately 45 min per subject.

### 2.3. Data Preprocessing

The recorded data of each stimulus type was then epoched into trials from −0.5 to +4 s centered on stimulus onset. Through visual inspection, trials containing high amplitude recorded artifacts were then removed from the MEG data. Trials in which the subject did not comply with the paradigm timing e.g., “subject spoke before the cue to articulate,” were also discarded. After data preprocessing a total of 3, 046 valid trials were retained out of 4, 000 (8*subjects* × 5*phrases* × 100*trials*) recorded trials with an average of 75 trials per phrase per subject. These valid trials were then low-pass filtered below 250 Hz with a 4th order Butterworth filter for further analysis. For this study, only gradiometer sensors were considered for decoding considering their effectiveness in noise reduction and representation of the stimuli based activation. Out of 204 gradiometer sensors, four sensors showed high channel noise during data collection from different subjects. Further, in case of some subjects, one or two more sensors showed artifact like irregularities. In total, data from eight sensors were excluded. In other words, data from 196 sensors were used for analysis.

### 2.4. Wavelet Analysis

Even though the signals were checked rigorously for artifacts, further presence of noise would hamper the characteristics of true brain oscillations. To address this issue, researchers typically employ one or several denoising algorithms (Haumann et al., 2016) including short-time Fourier transform, temporal signal source separation (t-SSS), principal component analysis (PCA), independent component analysis (ICA), and wavelet transform, etc. In particular, wavelets have been widely used for the denoising of bio-signals including MEG (Dash et al., 2018c). Wavelets express a signal as a linear combination of a distinct set of functions, obtained by shifting and scaling a single function (mother wavelet). Although the preprocessed MEG signals were in 1 kHz sampling frequency range, functional brain oscillations are believed to exist up to the high-gamma frequency range (< ~125 Hz) (Ahnaou et al., 2017). Thus, we employed the Daubechies (db)-4 wavelet with a 7 level decomposition to perform discrete wavelet transform (DWT) for denoising and decomposing the MEG signals to specific neural oscillations. Mathematically, a signal s with a seven level wavelet decomposition can be represented as:

(1) $$\begin{array}{l} s=d_{1}+d_{2}+d_{3}+d_{4}+d_{5}+d_{6}+d_{7}+a_{7} \end{array}$$

**Figure 3:**
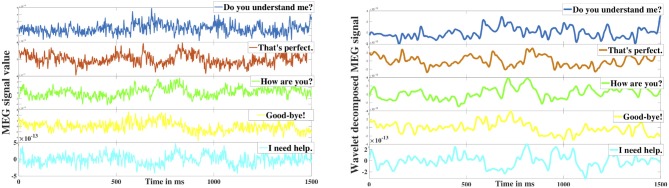
Wavelet denoising. The left image represents the 5 raw MEG signals obtained from a sensor corresponding to the five different phrases. The right image represents the corresponding denoised signals.

Here, *d*_1−7_ are the detail coefficients whereas *a*_7_ is the low-frequency approximation coefficient. The signal is decomposed in such a way that in each level the signal disintegrates into two components (details and approximation) such that the detail component carries the high-frequency (upper half) element whereas the approximation component contains the low-frequency (lower half) oscillations. In this case, *d*_1_ and *d*_2_ are the high-frequency signals with the frequency range 250−500 and 125−250 Hz respectively which were discarded as noise. The effectiveness of the proposed db-4 based denoising can be observed in Figure 3 which shows the comparison of raw signal vs. denoised signal after reconstruction (<125 Hz), Since it has been repeatedly shown that the neural information is encoded up to high gamma frequency bandwidth, removal of high-frequency components (>125 Hz) was necessary. After removing *d*_1_ and *d*_2_, the reconstructed signals from the remaining detail frequency components *d*_3−7_ represented the high-gamma (62–125 Hz), gamma (31–58 Hz), beta (16–30 Hz), alpha (8–16 Hz), and theta (4–8 Hz) frequency bands of the neural signal. The reconstructed approximation signal from *a*_7_ was the low-frequency delta band oscillation (0.1–4 Hz).

## 3. Decoding Approaches

In this study, we performed a five-class classification task where each class corresponded to one phrase. Considering the tremendous cognitive variance across subjects (Dash et al., 2019c), only subject dependent decoding was performed, where training and testing data were from the same speakers (but unique). The classification task was performed on each of the four whole data segments (i.e., pre-stimuli, perception, preparation/imagination, and production/articulation). We leveraged two machine learning algorithms including a classic ANN as the baseline and the latest CNNs (i.e., AlexNet, ResNet, Inception-ResNet). The input to ANN was the root mean square (RMS) features of the denoised and decomposed MEG signals from each data segment. The input to CNNs was scalogram images generated from the denoised MEG signals of the whole data segments. Each of these methods is briefly described below.

### 3.1. Artificial Neural Network (ANN)

**Figure 4:**
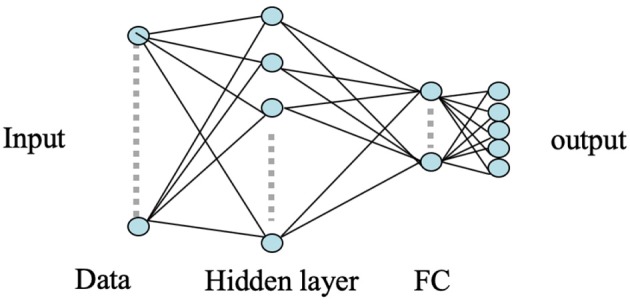
ANN architecture. Here the circles represent the nodes whereas the arrows represent the connections between the nodes. The input layer is an 1, 176 dimensional feature vector. The hidden layer consists of 256 nodes and the remaining layers are of 5 nodes.

ANNs have been widely used for pattern classification problems to model a set of inputs leading to corresponding target outputs. The architecture of ANN is characterized by multiple connected nodes or neurons for functional processing. Considering its robust and efficient non-linear computational modeling, we used a shallow ANN as our baseline approach to classify the MEG acquired neural responses of the brain for the five respective stimuli. The input to the ANN classifier was the concatenated RMS features obtained from each of the six neural oscillation signals, high-gamma, gamma, beta, alpha, theta, and delta. A total of 196 gradiometer sensors (204 gradiometers—8 discarded due to noise) were considered for analysis. Thus, the input feature dimension of the ANN was 1, 176 (6 frequency bands × 196 sensors). A variety of statistical features (mean, median, standard deviation, quartiles, tertiles, energy, windowed energy, cross-correlation matrix) were first extracted and examined for the statistically significant difference across the 5 classes. RMS turned out to be the best feature which was significantly different across classes (1-way ANOVA, followed by Tukey; *P* < 0.001). Feature combination was not explored since the dimension of a single type of feature was already very large (1, 176). A single hidden layer consisting of 256 number of hidden nodes with randomized weights was used during the initialization of the ANN model. A five-dimensional sigmoid activation function was connected after the hidden layer to transform the learned weights into a non-linear hyper-dimensional space. A five-dimensional fully connected softmax layer was used after sigmoid which was further connected to a five-dimensional fully-connected (FC) output classification layer to represent the cross-entropy of the five phrases. The weights of the nodes in the hidden layer of the ANN were updated via back-propagation using the stochastic gradient descent algorithm. The architecture of the used shallow ANN model is shown in Figure 4.

We used a coarse-to-fine hypermeter tuning strategy for tuning the learning rate with the range of values: 0.1, 0.01, 0.001, and 0.0001 on validation data, where 0.01 yielded the best performance and was used in the experiment. The data was divided into three parts as train, validation, and test such that train data consisted of 70% of the whole data whereas the test and validation data consisted of 15% each of the whole data. Our previous finding on determining the optimal number of trials for speech decoding with ANN (Dash et al., 2018c) has suggested that a total of 40 trials are sufficient for speech decoding after which the performance saturates. Hence, the traditional data split (70%−15%−15%) of train-validation-test was performed. Further, to avoid biased split, we performed ANN training on three separate random splits to find the average performance. Data overfitting was checked with the validation data by ensuring the early stopping of the training when the model started to generalize the data. A continuous increase in validation loss for more than 6 epochs was considered as the threshold for data overfitting. Although the maximum number of epochs were set to 100, as the data size was small, data overfitting started to occur even after an average of 35 epochs. Further, we have experimented with various combinations of hidden layer nodes to train the model to find the optimal number of nodes to train the MEG data. We tuned with various 64 × nodes (i.e., 64, 128, 192, 256, 320, 384, 448, 512, 640, and 1, 024 nodes) and observed an increase in validation accuracy from 64 to 256 and then the validation accuracy saturated after 256 nodes until 512 nodes. Early data over-fitting resulted while training with more than 512 nodes in the hidden layer.

### 3.2. Convolutional Neural Networks (CNNs)

**Figure 5:**
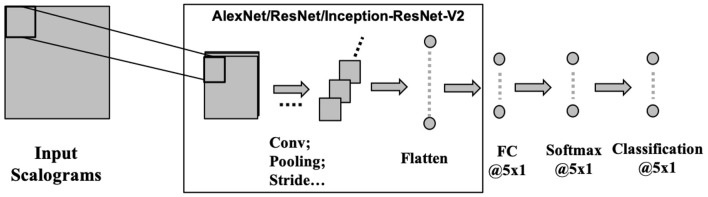
CNN architecture. Three pre-trained deep CNNs were used with the final layers replaced by fully connected, softmax, and classification layer each with 5 nodes to suit for a 5-phrase classification problem.

CNNs operate on the data by applying convolution operation on a selected receptive field. CNN makes the implicit assumption of the inputs to be images, which allows for encoding of certain properties into the architecture. CNNs are scale and shift-invariant based on their shared weight and translation invariance characteristics. Typically, a CNN architecture is formed by a stack of distinct layers (convolution, pooling, and activation) that transform the input data to an output volume with relevant class scores through a differentiable function. Here, we have used three recent deep convolutional neural networks namely AlexNet (Krizhevsky et al., 2017), ResNet101 (Wu et al., 2018), and Inception-ResNet-v2 (Szegedy et al., 2016) to evaluate the effectiveness of CNN for speech decoding (Figure 5). Each of the three architectures has been popularly used as classifiers for their high-performance achievement. These deep ConvNets are pre-trained with more than a million images of 1, 000 categories from the ImageNet database (Russakovsky et al., 2015) to learn rich features from the images.

#### 3.2.1. AlexNet

AlexNet was the first deep CNN to be introduced which increased the accuracy with a very high stride compared to the then traditional approaches. It consists of five convolution layers and three fully connected (FC) layers. The kernels (filters) employed in this CNN architecture are of 11 × 11, 5 × 5, and 3 × 3 sizes and has rectified linear unit (ReLU) activation function after each convolution operation. ReLU along with dropouts were first introduced in this architecture which make AlexNet significantly faster and over-fit voided. Further, with dropouts, neurons are randomly chosen and are switched off. This restricts the neurons to coadapt and hence they learn meaningful features, independent of other neurons.

#### 3.2.2. ResNet

ResNets introduced residual modules in the architecture which solved the degradation problem (naive addition of deeper layers leading to high training error) during the training of deeper networks. These modules create a direct pathway between input and output and learn the features on top of the available input. The residual networks were shown to be easily optimized and can gain accuracy with a significantly deeper architecture. In other words, residual modules can be thought of as shortcut connections for identity mapping. This architecture consists of 101 layers with largely 3 × 3 filters. The other attribute of this architecture is the use of global average pooling which is discussed to contribute to better accuracy since it's more native to the convolutional structure and more robust to the spatial translations of the input.

#### 3.2.3. Inception-ResNet-v2

Inception-ResNet-v2 is the second version of the combined Inception and ResNet architecture based on the idea of Microsoft ResNet to integrate residual modules on top of Inception architecture. This network has achieved one of the best performances in the ILSVRC classification task (Russakovsky et al., 2015). This ConvNet is 164 layers deep consisting of one Inception-v4 with three residual networks. The advantage of the Inception network is that here the inputs go through 1 × 1, 3 × 3, and 5 × 5 kernels simultaneously with max-pooling which are then concatenated to form the output. Hence, there is no need of deciding on the filter size at different layers. Further, the addition of residual networks accelerates the training of the Inception-v4 network.

#### 3.2.4. Features for CNNs

In this experiment, two types of features were used to validate the efficacy of CNN in speech decoding. The first of these were spectral-temporal features extracted from the MEG signals, that were the scalogram images of the gradiometer signals which consist of the multi-scale variation of spectral and temporal features. The second was spatial-spectral-temporal features where we embedded the spatial information (sensors) of the corresponding gradiometers in the images. Color scalogram images were generated from the two types of features and were used as the input to the three CNN architectures.

##### 3.2.4.1. Spectral-temporal features

**Figure 6:**
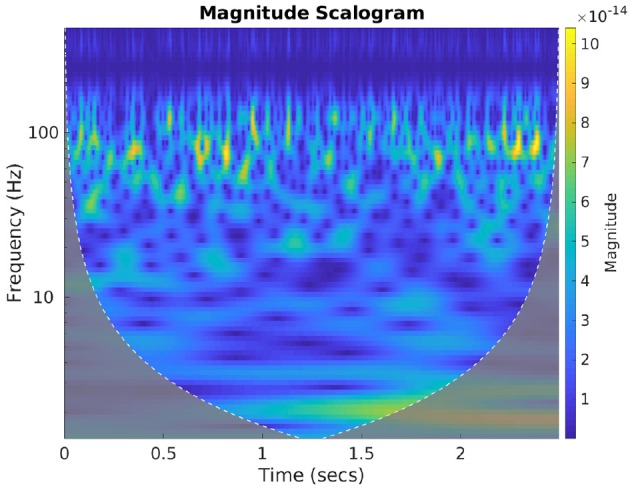
Scalogram plot of neural signal. The image represents the cone of influence (COI) plot of the scalogram representation of the brain signal obtained from a sensor near Broca's area. This is when the subject was speaking “Do you understand me.” The color bar represents the change in energy of various CWT coefficients obtained with Morlet wavelet.

Spectral features of neural signals carry important latent attributes of neural response (Halme and Parkkonen, 2016). To benefit from the frequency information of the brain activity signals we computed the wavelet scalograms of the denoised MEG signals by performing continuous wavelet transform (CWT) with Morlet wavelets. For this, the db-4 decomposed signals were first reconstructed back up to the 2nd level to accommodate all the neural oscillations (up to high-gamma frequency bandwidth). CWT generates an overcomplete representation of the signal under analysis by convolving the input data with a set of functions obtained by scaling and translating the mother wavelet (here Morlet wavelet) across various scales. The energy values of the CWT coefficients are represented as scalogram images which are extremely useful in conveying the spectral-temporal characteristics of a signal (Lilly and Olhede, 2009). Morlet wavelet has been shown to be highly effective in characterizing the MEG signal features (Tadel et al., 2011), hence we used this wavelet to compute the scalograms. Figure 6 gives an exemplary scalogram image of the neural signal corresponding to a sensor approximately near to Broca's area while a subject is articulating “do you understand me.” The scalogram images for each sensor signal during each stage were generated for all the valid trials and then resized to the specific size based on the requirement of the corresponding CNN architecture (AlexNet, ResNet, and Inception-ResNet-v2) and trained with each scalogram image as a sample. The evaluation of classification accuracy with this feature was done on single-trial level by computing the average cross-entropy score obtained from the scalogram images of all 196 sensors within a single-trial.

##### 3.2.4.2. Spatial-spectral-temporal features

**Figure 7:**
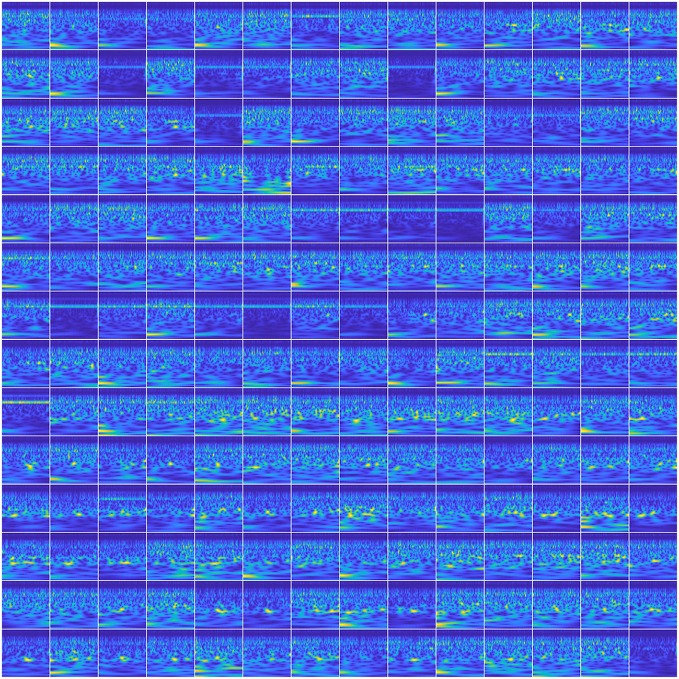
Scalogram matrix of neural signal. The image represents a 14 × 14 matrix of scalogram images to represent the whole brain dynamics in a single image. This is when the subject was speaking “Do you understand me” for a single trial.

To utilize the spatial information of the MEG signals in the input images, we created a 14 × 14 matrix of scalogram images (obtained from 196 sensors) within a single image (see Figure 7). With this representation, for a single trial, the spatial (location) information of all the sensors was encoded within a single image. With this feature representation, the number of input images to be trained with the networks became the same as the number of trials which is about 50 per phrase per subject. Training these deep ConvNets requires a considerably higher number of inputs for proper training. Hence, we leveraged a commonly-used data augmentation approach to address this issue. Data augmentation as a self-regularizer has been demonstrated to be effective in machine learning (Shorten and Khoshgoftaar, 2019) particularly for small-data size problems. A linear positive shift of both 100 and 200 ms was performed on the signals and then their scalogram images were generated. Since the average reaction time of the subjects for speech production was about 250 ms, this linear shift mechanism also helped in compensating for the changes in the articulation onset along with inducing variability in the input images. With this, the data size was increased to three times larger than the original and was sufficient for the training of the same three CNNs described above. To avoid possible false positives, data augmentation was implemented within each set (training, validation, or testing). In other words, a trial and its augmented versions were always under the same group (training, validation, or testing). Data augmentation for the other CNN approach (with spectral-temporal features) and the baseline approach was not needed since the training data was sufficient as observed with low variance error. Further, for ANN analysis, we have previously shown that after 40 trials, the decoding performance saturates (Dash et al., 2018c), hence, data augmentation was not necessary.

#### 3.2.5. Experimental Setup for CNNs

As per the input requirement, for AlexNet the images were resized to 227 × 227 × 3, for ResNet101 to 224 × 224 × 3 and then for Inception-ResNet-v2 the input images were of size 299 × 299 × 3. The third dimension (3) represents the three (RGB) colored channels. Since, these networks have been trained for a 1, 000 class image classification problem, to tune these networks for our 5 class classification, the last few layers were modified, keeping the initial layers untouched. For AlexNet, the last three layers were replaced by an FC layer, a softmax layer, and a classification layer each with five nodes. Similarly, we used this five-dimensional FC layer and softmax layer to replace the last two layers of ResNet101 (fc1000 and classificationLayer-Predictions) and of Inception-ResNet-v2 (Predictions and ClassificationLayer-predictions).

Figure 5 shows the architecture for implementing the deep CNNs. For unbiased comparison, the learning rate for these three networks was fixed at 0.0001. The weight-learn-rate-factor and the bias-learn-rate-factor in the final fully-connected (FC) layer were increased to 20 for faster learning in the new layers than the transferred layers. For all the networks, Adam optimizer, a minibatch size of 64, validation frequency and validation patience of 6, a maximum epoch of 60 and gradient clipping was used. The rest of the hyperparameters were kept at their default values of the respective architectures. The same data partitioning (70%-training, 15%-validation, and 15%-testing) approach was employed here as well. The testing data were completely unseen (without containing any augmented version of the training or validation trials) and hence were new to the model. Only validation data were used for hyperparameter tuning and overfitting checking in the training stage. The CNNs were trained on a 7-GPU parallel computing server running on Linux (Ubuntu 16.04) platform using Keras imported to Matlab 2018b.

## 4. Results

### 4.1. Performances of the Decoding Approaches

**Figure 8:**
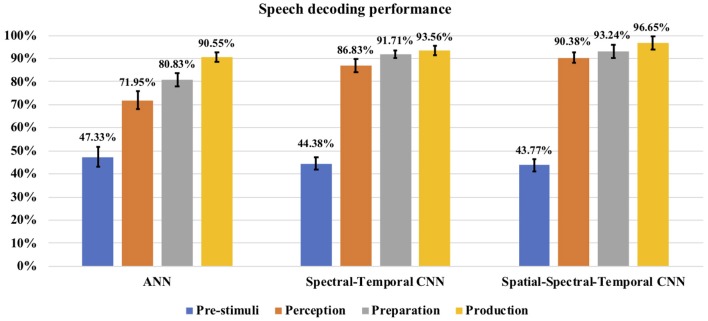
Speech decoding accuracy. The bar graph represents the speech decoding accuracy obtained with different classifiers. For CNN the results with AlexNet are reported. The error bars represent the standard deviation in decoding accuracy across subjects.

The classification accuracy was computed during each stage for each subject and the average classification accuracy across the eight subjects with each method can be seen in Figure 8. The decoding performances during all the four stages (pre-stimuli, perception, preparation/imagination, articulation) obtained with ANN or CNNs were significantly higher than the chance level accuracy (30%) (1-tail *t*-test, *p* < 0.05 for all). With the shallow ANN (the baseline approach), the average classification accuracy during the articulation stage was satisfactorily high (90.55 ± 2.11%), but not for perception (71.95 ± 3.97%) and imagination stage (80.83 ± 3.00%) (Figure 8). Both of the approaches using CNN classifier [spectral-temporal CNN (ST-CNN) and spatial-spectral-temporal CNN (SST-CNN)] outperformed the baseline (ANN) in terms of the average decoding accuracy during perception, imagination, and articulation. The average classification accuracies obtained with ST-CNN during perception, preparation, and production were 86.83 ± 2.93%, 91.71 ± 1.67%, and 93.56 ± 1.92%, respectively, whereas, with SST-CNN the accuracies were 90.38 ± 2.28%, 93.24 ± 2.87%, and 96.65 ± 2.88%. The differences between the decoding performances of ANN and CNN were statistically significant, which was observed via the pairwise comparison of the decoding performances with ANN, ST-CNN, and SST-CNN (2-tail *t*-test, *p* < 0.05, for all possible pairs). The highest *p*-value among all pairwise comparisons was 0.0099 when the decoding performance of ANN and ST-CNN was compared during the production stage. Among the two approaches involving CNN, spatial-spectral-temporal-CNN (SST-CNN) performed better than the spectral-temporal-CNN (ST-CNN) in terms of average decoding accuracies. A 2-tail *t*-test comparison of decoding performances between these two methods resulted in a significant difference between all pairs (*p* < 0.05), except between spectral-temporal and spatial-spectral-temporal features with CNN during the preparation stage (*p* = 0.2135). For the pre-stimuli stage there was no significant difference between the performances of all the three methods (2-tail *t*-test, *p* > 0.05).

**Figure 9:**
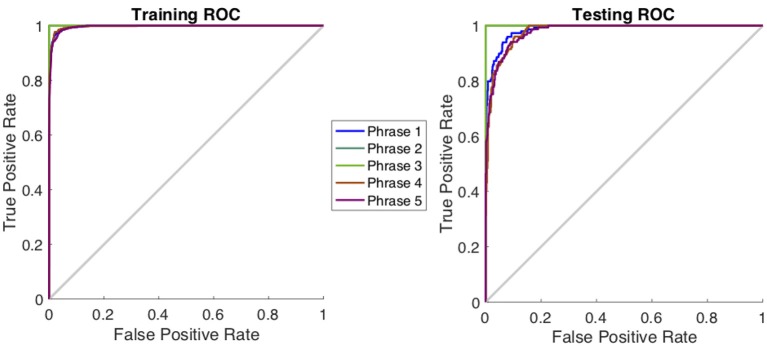
Receiver operating characteristics curve for one subject during the classification of phrases in the articulation stage using AlexNet. The plot represents the variation between true positive rate (sensitivity) and true negative rate (1-specificity) to indicate the classification performance. For this curve average AUC >0.96 indicating a very high classification performance.

Table 1 shows a comparison of the three specific CNN architectures, where AlexNet slightly outperformed ResNet101 and Inception-ResNet-v2 in terms of decoding accuracy. Although it has been shown that Inception-ResNet-v2 performs better with the ImageNet database, in case of MEG scalograms it was slightly different. We believe that the choice of higher initial kernel size (11 × 11) in the AlexNet architecture might have contributed to better performance. In the scalogram images, the features are represented with energy blobs, thus a higher initial kernel size might have helped produce better feature extraction. Nevertheless, the performances of the three ConvNets were quite similar with a standard deviation of < 3% (Table 1). This further strengthens the efficacy of CNNs for neural speech decoding. To illustrate the details of the classification performance via the best decoder (AlexNet), Table 2 gives the confusion matrix obtained by combining the results from all the test sets across all subjects during articulation, where the primary diagonal numbers are the correctly classified sample. The average number of misclassified samples per phrase was about 12 in the combined test set (1, 382) of 8 subjects, i.e., 0.9% misclassification per phrase. Further, the receiver operating characteristics was plotted for each classification to observe the variation of true positive rate (sensitivity) with false positive rate (1-specificity) (Figure 9: Exemplary ROC curve for overt speech decoding with SST-CNN using AlexNet during training and testing) to validate the classification. Figure 9 clearly shows the validation of the classification performance across classes with a very high area under the curve (AUC).

**Table 1:** Performance comparison of AlexNet, ResNet101, and Inception-ResNet-v2 in terms of decoding accuracies with spatial-spectral-temporal features.

**CNN architecture**	**Pre-stimuli (%)**	**Perception (%)**	**Preparation (%)**	**Production (%)**
AlexNet	43.77	90.38	93.24	96.65
ResNet101	42.61	84.75	87.78	92.36
Inception-ResNet-v2	42.52	87.98	91.66	94.49
Mean	42.96	87.70	90.89	94.50
STD	0.70	2.82	2.81	2.14

*The test accuracies are averaged over 3 independent runs across subjects*.

**Table 2:** Test confusion matrix for speech decoding accumulated across all subjects during articulation stage.

		**Predicted**					
		**Do you understand me**	**That's perfect**	**How are you**	**Good bye**	**I need help**	**Accuracy (%)**
	Do you understand me	1,332	12	19	11	8	96.38
	That's perfect	10	1,338	14	11	9	96.82
	How are you	21	8	1,331	12	10	96.31
	Good bye	12	15	12	1,337	6	96.74
True	I need help	7	12	14	7	1,342	97.11
	Average:						96.67

*A total of 1, 382 test trials have been taken (combined across 8 subjects)*.

### 4.2. Performances During Pre-stimuli, Perception, Imagination, and Production

Among the four stages (i.e., pre-stimuli, perception, imagination, and production), best classification accuracy was always during production, then imagination, followed by perception. Comparing classification accuracies of all the four stages (i.e., pre-stimuli, perception, preparation/imagination, production; in pairs), irrespective of classifiers, all the results were significantly different (2-tail *t*-test, *p* < 0.05), except in one case, where no significant difference was observed while comparing between preparation and production stage using spectral-temporal CNN. A slightly higher *p*-value than the desired confidence (*p* = 0.0589) was observed. During imagination, the accuracy of average speech decoding was above 91% in both cases of feature representations (ST-CNN and SST-CNN), which indicates that the information processing in the brain occurs prior to articulation. Also, with the speech perception segment, a high level (around 87–90%) accuracy was obtained, which is not surprising. This provides further evidence in the literature that decoding speech perception from the MEG signal is viable. Theoretically, the decoding accuracy for the pre-stimuli segment should be at the chance level which is about 30% for N = ~300 (~ 60 trials × 5 classes) (Combrisson and Jerbi, 2015) (20% is for ideal population size), as there was no stimulus or task during this stage. However, the speech decoding accuracy obtained in this stage with all the classifiers were significantly higher than the chance level (1-tail *t*-test, *p* = 0.00013).

## 5. Discussion

### 5.1. Comparison of Decoding Approaches

Overall, we found that CNNs with spatial-spectral-temporal features performed better than CNNs using only the spectral-temporal features, and that both CNN approaches outperformed the ANN classifier. We used only a single value (RMS) feature from one sensor for the ANN classifier. Temporal information was not well represented here, which may explain the lower accuracy compared to the CNNs and indicate the usefulness of temporal information for decoding. In the current experiment, the ANN feature dimension was 1, 176 which was higher than the number of samples for training (about 250 per subject across 5 phrases). A dimension reduction strategy prior to the ANN training may improve the performance. There has long been evidence regarding the role of neural oscillations in brain function (Buzsáki and Draguhn, 2004; Formisano et al., 2008), and spectral features of the brain activity signals almost certainty carry more information than the integrated energy of MEG signals. Thus, it is not surprising that we found better classification accuracy using the spectral-temporal features (scalograms) based CNN classifier compared to the ANN classifier. However, a clear interpretation of what aspects of the scalograms led to better performance is not straight forward, because of the inherent differences in the feature sets, in content and dimension. For our third approach, we added spatial information on top of spectral-temporal features by combining the scalograms of all the sensors into one image, thereby representing the neural dynamics across the whole brain. The improved results obtained with this approach (SST-CNN) indicate that CNNs were indeed able to better utilize the spatial, spectral, and temporal information from the MEG signals and for learning the appropriate feature set for the decoding tasks. The use of the scalogram images to represent the whole brain dynamics is a novel strategy to leverage the efficiency of CNNs. The same strategy can be applied while decoding with EEG or ECoG channels as well. It is possible that the data augmentation used in the SST-CNN approach might have contributed to better performance by inducing variability in the data besides increased data size for better CNN training. As mentioned previously, we did not use data augmentation for the other CNN approach (ST-CNN), because the data were sufficient for training it. We believe that using data augmentation in ST-CNN might also increase its performance. However, the training would be extremely time-consuming.

One approach may be to transform the RMS features of 1, 176 dimension to a matrix (of size 14 × 28 × 3 or equivalent) and then feed to the 2D-CNNs for classification. However, there are numerous possible ways to construct such a matrix. The other way of transforming the images into vectors to be used as input to ANN is also not ideal as the feature vector dimension would be huge. Thus, here we did not intend to compare the features rather we compared the approaches used for decoding. It should be noted that this study evaluated subject-dependent classification performance, where training and testing data were from the same subjects (but unique). Hence, the features learned via CNN might be subject-specific and may not generalize across the population. Performing speaker-independent decoding is extremely challenging considering the cognitive variance across subjects. Subject normalization/adaptation based strategies are needed to be assessed to address the subject-independent decoding problem in the future (Dash et al., 2019d).

### 5.2. Contribution of Wavelets in Decoding

In the current study, we used discrete (DWT) and continuous (CWT) wavelet transforms as a means to both reduce the influence of noise and extract neural features from the neuromagnetic signals. There are numerous studies showing wavelet decomposition to be an effective method for noise and artifact reduction, including those generated by muscles signals (Vialatte et al., 2008; Klados et al., 2009; Safieddine et al., 2012; Harender and Sharma, 2017). Indeed, in a previous study using a subset of the current data, we observed an increase in SNR of the neural signals after DWT (Dash et al., 2018c).

Likewise, decomposing the signal into discrete neural brainwaves may have assisted in producing robust feature representations. Comparing the current ANN decoding results—for three subjects—with an earlier pilot study (Dash et al., 2018d), we observed that wavelet decomposition accounts for an average decoding accuracy improvement of roughly 2%. While this may a be modest improvement, our ANN feature (RMS) integrates over the temporal domain effectively, removing temporal information that is well known to reflect specific functional processes (Andreou et al., 2015). Specific frequencies have been used in the past for decoding (Chi and John, 2011), so it stands to reason that the temporal information in our novel scalogram approach may have significantly contributed to decoding efficiency in the present work.

### 5.3. Comparisons in Decoding Imagination and Articulation

The observation that spoken information can be more efficiently retrieved directly from the brain during articulation than perception and preparation (imagination) could be because of the involvement of motor cortex (for the movement of articulators) and auditory cortex (for auditory feedback). One may argue that the articulatory (jaw) motion artifact that remained in the brain signals after denoising also contributed to the higher decoding accuracy during speech production than imagination. However, our previous study has suggested that MEG signals have more information than just jaw motion signal itself in speech decoding (Dash et al., 2018d). We found that by combining MEG and jaw motion signals, better decoding performance can be achieved than using each of them separately. Of note is that our current protocol has 1–1.5 s delay after articulation ends (before the next trial starts). However, the effect of speech processing may continue in the brain (carryover) even many seconds after the articulation ends. From the decoding perspective, however, we considered this to be a challenge since previous trials could possibly corrupt the current trial's signals at least during the pre-stimuli and possibly the perceptual phase. We interpret our results to be robust despite the possible interference, and in support of a hypothesis that it is possible to decode spoken phrases from non-invasive signals.

Another concern may be that the phrase duration information might contribute to the decoding of spoken phrases, for example, “good-bye” and “do you understand me” have considerably different lengths. While we think this is a caveat of our experimental design, there is reason to be optimistic that the main contribution is from phrase information, not just duration. As shown in Table 2, the decoding error (mislabeling) between “Do you understand me?” and “Good-bye” (11) was not considerably different from the mislabeling between “I need help” and “Good-bye” (7), which are of similar duration. In fact, all the mislabeling across the five phrases were similar. On the other hand, duration is an important feature for speech recognition and is commonly used in decoders for phonemes. In future studies, we plan to control the phrase duration to better understand the role of duration in phrase/phoneme decoding.

High imagined phrase decoding performance opens up the possibility of direct brain to text mapping applications for completely paralyzed patients by retrieving the intended speech from the brain without needing articulation. Although, it can be argued that, participants could have already activated vocal muscles during imagination, the inherent time-locked experimental design prevents this before the start of the articulation stage. Our experimental protocol was designed to collect both imagined and overt speech consecutively within a trial. After prompting the subject with the stimulus, only 1 s was given for the subject to imagine the pronunciation of the phrase, after which the subject articulated the phrase. This limited 1 s duration attempted to ensure the behavioral compliance of the subject to imagine only that phrase. It is extremely difficult to verify whether the subjects actually performed the task of imagination in the traditional setting of imagined speech data acquisition (Cooney et al., 2018). Hence, we collected both imagined and overt speech in a single trial with a limited duration for the imagination segment for behavioral control. In our design, 1 s may be sufficient for imagination because the average production time of the longest phrase (Do you understand me?) was 0.97 ± 0.08 s across trials, and considering that imagined speech may be faster than overt speech (Indefrey and Levelt, 2004; Oppenheim and Dell, 2008). Nevertheless, our paradigm may have a combination of both preparation and imagination within the 1 s duration. Based on the extant literature, phonological representation is shown to be activated during orthographical language processing and the preparation (pre-speech) (Cooney et al., 2018). The extent to which these phonological processes are existing in our preparation/imagination stage is not clear and would require specifically designed studies to dissociate them, if possible. Nonetheless, the high accuracy obtained during the imagined phrase segment is encouraging and provides strong support of the existence of sufficient information for fast decoding of intended speech for real-time BCI.

### 5.4. Toward a Next-Generation Speech-BCI

The objective of this study was to demonstrate the possibility of direct speech decoding from neural signals, which is to support the development of the next generation, more efficient, speech decoding-based BCIs. Our results have shown the feasibility to decode speech directly from MEG signals. Although we focused on a small set of stimuli (five phrases) in the early stage of this study, future studies will focus on decoding an open vocabulary set (any phrases). Another barrier for the development of a speech-BCI is that MEG is currently not suitable for this application due to its high cost, size, and immobility. Encouraging recent work on wearable, OPM-based MEG systems (Boto et al., 2018; Roberts et al., 2019; Zetter et al., 2019) has shown that it is possible to build portable MEG machines with a significantly reduced cost and size (equivalent to the size of a helmet). This technological advance opens the possibility for utilizing OPM-based MEG as a speech-BCI to potentially restore communication for locked-in patients.

## 6. Conclusions

In this study, we demonstrated the possibility of decoding imagined and spoken phrase directly from non-invasive neural (MEG) signals using ANN and CNNs. We observed that speech decoding accuracy was the best during the speech production stage over other stages. However, even during the speech preparation (imagination) stage, the accuracies were very high, which suggests the feasibility for decoding intended or covert speech for the next-generation BCIs. Three state-of-the-art CNN architectures were used to provide evidence in support of the efficacy of CNNs in speech decoding over ANN. In addition, a unique representation of spatial, spectral and temporal features to represent the whole brain dynamics was found to be crucial in this neural speech decoding experiment. This study was only performed on healthy subjects. A further investigation on neural speech decoding from locked-in/ALS patients is needed to establish MEG as a potential device for the development of next generation, faster BCIs.

## Data Availability Statement

The data is currently not ready for distribution but is under the plan to be ready in the future. Codes can be obtained from the corresponding authors upon request.

## Ethics Statement

The study involving human participants was reviewed and approved by The University of Texas at Dallas (IRB# 15-92), which covered the data collection at the Cook Childrens Hospital (Fort Worth, TX) through a service, and by The University of Texas at Austin (IRB# 2015-09-0042). All participants provided their written informed consent before participating in this study.

## Author Contributions

DD implemented the algorithms and drafted the manuscript. PF and JW designed the experimental paradigm for data collection. DD, PF, and JW interpreted the results and performed subsequent editing.

### Conflict of Interest

The authors declare that the research was conducted in the absence of any commercial or financial relationships that could be construed as a potential conflict of interest.

## References

[B1] Ackermann H. (2008). Cerebellar contributions to speech production and speech perception: psycholinguistic and neurobiological perspectives. Trends Neurosci. 31, 265–272. 10.1016/j.tins.2008.02.011 18471906

[B2] Ahnaou A. Huysmans H. Casteele T. V. D. Drinkenburg W. H. I. M. (2017). Cortical high gamma network oscillations and connectivity: a translational index for antipsychotics to normalize aberrant neurophysiological activity. Transl. Psychiatry 7, 1–14. 10.1038/s41398-017-0002-9 29249806 PMC5802558

[B3] Akbari H. Khalighinejad B. Herrero J. L. Mehta A. D. Mesgarani N. (2019). Towards reconstructing intelligible speech from the human auditory cortex. Sci. Rep. 9:874. 10.1038/s41598-018-37359-z 30696881 PMC6351601

[B4] Andreou L.-V. Griffiths T. D. Chait M. (2015). Sensitivity to the temporal structure of rapid sound sequences—An MEG study. NeuroImage 110, 194–204. 10.1016/j.neuroimage.2015.01.052 25659464 PMC4389832

[B5] Angrick M. Herff C. Mugler E. Tate M. C. Slutzky M. W. Krusienski D. J.. (2019). Speech synthesis from ECoG using densely connected 3D convolutional neural networks. J. Neural Eng. 16:036019. 10.1088/1741-2552/ab0c59 30831567 PMC6822609

[B6] Anumanchipalli G. Chartier J. F. Chang E. (2019). Speech synthesis from neural decoding of spoken sentences. Nature 568, 493–498. 10.1038/s41586-019-1119-1 31019317 PMC9714519

[B7] Birbaumer N. (2006). Brain-computer-interface research: coming of age. Clin. Neurophysiol. 117, 479–483. 10.1016/j.clinph.2005.11.002 16458595

[B8] Boto E. Holmes N. Leggett J. Roberts G. Shah V. Meyer S. S.. (2018). Moving magnetoencephalography towards real-world applications with a wearable system. Nature 555, 657–661. 10.1038/nature26147 29562238 PMC6063354

[B9] Brigham K. Vijaya Kumar B. V. K. (2010). “Imagined speech classification with EEG signals for silent communication: a preliminary investigation into synthetic telepathy,” in 2010 4th International Conference on Bioinformatics and Biomedical Engineering (Chengdu), 1–4.

[B10] Brumberg J. Wright E. Andreasen D. Guenther F. Kennedy P. (2011). Classification of intended phoneme production from chronic intracortical microelectrode recordings in speech motor cortex. Front. Neurosci. 5:65. 10.3389/fnins.2011.00065 21629876 PMC3096823

[B11] Brumberg J. S. Nieto-Castanon A. Kennedy P. R. Guenther F. H. (2010). Brain-computer interfaces for speech communication. Speech Commun. 52, 367–379. 10.1016/j.specom.2010.01.001 20204164 PMC2829990

[B12] Buzsáki G. Draguhn A. (2004). Neuronal oscillations in cortical networks. Science 304, 1926–1929. 10.1126/science.1099745 15218136

[B13] Cao B. Kim M. van Santen J. Mau T. Wang J. (2017). “Integrating articulatory information in deep learning-based text-to-speech synthesis,” in Proceedings of Interspeech 2017 (Stockholm), 254–258.

[B14] Cheyne D. Ferrari P. (2013). MEG studies of motor cortex gamma oscillations: evidence for a gamma “fingerprint" in the brain? Front. Hum. Neurosci. 7:575. 10.3389/fnhum.2013.00575 24062675 PMC3774986

[B15] Chi X. John H. (2011). EEG-based discrimination of imagined speech phonemes. Int. J. Bioelectromagn. 13, 201–206. Available online at: http://www.ijbem.org/volume13/number4/201-206.pdf

[B16] Cireşan D. C. Meier U. Masci J. Gambardella L. M. Schmidhuber J. (2011). “Flexible, high performance convolutional neural networks for image classification,” in Proceedings of the Twenty-Second International Joint Conference on Artificial Intelligence - Vol. 2, IJCAI'11 (Barcelona: AAAI Press), 1237–1242.

[B17] Cohen D. Cuffin B. (1983). Demonstration of useful differences between magnetoencephalogram and electroencephalogram. Electroencephalogr. Clin. Neurophysiol. 56, 38–51. 10.1016/0013-4694(83)90005-6 6190632

[B18] Combrisson E. Jerbi K. (2015). Exceeding chance level by chance: the caveat of theoretical chance levels in brain signal classification and statistical assessment of decoding accuracy. J. Neurosci. Methods 250, 126–136. 10.1016/j.jneumeth.2015.01.010 25596422

[B19] Cooney C. Folli R. Coyle D. (2018). Neurolinguistics research advancing development of a direct-speech brain-computer interface. iScience 8, 103–125. 10.1016/j.isci.2018.09.016 30296666 PMC6174918

[B20] Cooney C. Folli R. Coyle D. (2019a). “Optimizing layers improves cnn generalization and transfer learning for imagined speech decoding from EEG,” in 2019 IEEE International Conference on Systems, Man and Cybernetics (SMC) (Bari), 1311–1316.

[B21] Cooney C. Korik A. Raffaella F. Coyle D. (2019b). “Classification of imagined spoken word-pairs using convolutional neural networks,” in Proceedings of the 8th Graz Brain Computer Interface Conference 2019 (Graz: Graz University of Technology), 338–343.

[B22] Dash D. Abrol V. Sao A. K. Biswal B. (2018a). “The model order limit: deep sparse factorization for resting brain,” in 2018 IEEE 15th International Symposium on Biomedical Imaging (ISBI 2018) (Washington, DC), 1244–1247.

[B23] Dash D. Biswal B. Sao A. K. Wang J. (2018b). “Automatic recognition of resting state fMRI networks with dictionary learning,” in Brain Informatics, eds Wang S. Yamamoto V. Su J. Yang Y. Jones E. Iasemidis L. Mitchell T. (Cham: Springer International Publishing), 249–259.

[B24] Dash D. Ferrari P. Heitzman D. Wang J. (2019a). “Decoding speech from single trial MEG signals using convolutional neural networks and transfer learning,” in 2019 41st Annual International Conference of the IEEE Engineering in Medicine and Biology Society (EMBC) (Berlin), 5531–5535. 10.1109/EMBC.2019.8857874 31947107

[B25] Dash D. Ferrari P. Malik S. Montillo A. Maldjian J. A. Wang J. (2018c). “Determining the optimal number of MEG trials: a machine learning and speech decoding perspective,” in Brain Informatics, eds Wang S. Yamamoto V. Su J. Yang Y. Jones E. Iasemidis L. Mitchell T. (Cham: Springer International Publishing), 163–172. 10.1007/978-3-030-05587-5_16 PMC6876632 31768504

[B26] Dash D. Ferrari P. Malik S. Wang J. (2018d). “Overt speech retrieval from neuromagnetic signals using wavelets and artificial neural networks,” in 2018 IEEE Global Conference on Signal and Information Processing (GlobalSIP) (Anaheim, CA), 489–493.

[B27] Dash D. Ferrari P. Malik S. Wang J. (2019b). “Automatic speech activity recognition from MEG signals using Seq2Seq learning,” in 2019 9th International IEEE/EMBS Conference on Neural Engineering (NER) (San Francisco, CA), 340–343.

[B28] Dash D. Ferrari P. Wang J. (2019c). “Spatial and spectral fingerprint in the brain: speaker identification from single trial MEG signals,” in Proceedings of Interspeech 2019 (Graz), 1203–1207.

[B29] Dash D. Wisler A. Ferrari P. Wang J. (2019d). “Towards a speaker independent speech-BCI using speaker adaptation,” in Proceedings Interspeech 2019 (Graz), 864–868.

[B30] Deng S. Srinivasan R. Lappas T. D'Zmura M. (2010). EEG classification of imagined syllable rhythm using hilbert spectrum methods. J. Neural Eng. 7:046006. 10.1088/1741-2560/7/4/046006 20551510

[B31] D'Zmura M. Deng S. Lappas T. Thorpe S. Srinivasan R. (2009). “Toward EEG sensing of imagined speech,” in Human-Computer Interaction. New Trends, ed J. A. Jacko (Berlin; Heidelberg: Springer Berlin Heidelberg), 40–48.

[B32] Formisano E. De Martino F. Bonte M. Goebel R. (2008). “Who” is saying “what”? Brain-based decoding of human voice and speech. Science 322, 970–973. 10.1126/science.1164318 18988858

[B33] Grill-Spector K. Henson R. Martin A. (2006). Repetition and the brain: neural models of stimulus-specific effects. Trends Cogn. Sci. 10, 14–23. 10.1016/j.tics.2005.11.006 16321563

[B34] Guenther F. H. Brumberg J. S. Wright E. J. Nieto-Castanon A. Tourville J. A. Panko M.. (2009). A wireless brain-machine interface for real-time speech synthesis. PLoS ONE 4:e08218. 10.1371/journal.pone.0008218 20011034 PMC2784218

[B35] Halme H.-L. Parkkonen L. (2016). Comparing features for classification of MEG responses to motor imagery. PLoS ONE 11:168766. 10.1371/journal.pone.0168766 27992574 PMC5161474

[B36] Harender and Sharma R. K. (2017). “EEG signal denoising based on wavelet transform,” in 2017 International Conference of Electronics, Communication and Aerospace Technology (ICECA), Vol. 1 (Coimbatore, TN), 758–761.

[B37] Hasasneh A. Kampel N. Sripad P. Shah N. Dammers J. (2018). Deep learning approach for automatic classification of ocular and cardiac artifacts in MEG data. J. Eng. 2018:1350692 10.1155/2018/1350692

[B38] Hashim N. Ali A. Mohd-Isa W.-N. (2018). Word-based classification of imagined speech using EEG,” in *Computational Science and Technology*, eds Alfred R. Iida H. Asri A. Ibrahim A. Lim Y. (Kuala Lumpur: Springer), 195–204.

[B39] Haumann N. T. Parkkonen L. Kliuchko M. Vuust P. Brattico E. (2016). Comparing the performance of popular MEG/EEG artifact correction methods in an evoked-response study. Intell. Neurosci. 2016:7489108. 10.1155/2016/7489108 27524998 PMC4972935

[B40] Herff C. Heger D. de Pesters A. Telaar D. Brunner P. Schalk G.. (2015). Brain-to-text: decoding spoken phrases from phone representations in the brain. Front. Neurosci. 9:217. 10.3389/fnins.2015.00217 26124702 PMC4464168

[B41] Huang Z. Yu T. (2019). “Cross-subject MEG decoding using 3D convolutional neural networks,” in 2019 WRC Symposium on Advanced Robotics and Automation (WRC SARA) (Beijing), 354–359.

[B42] Ikeda S. Shibata T. Nakano N. Okada R. Tsuyuguchi N. Ikeda K.. (2014). Neural decoding of single vowels during covert articulation using electrocorticography. Front. Hum. Neurosci. 8:125. 10.3389/fnhum.2014.00125 24639642 PMC3945950

[B43] Iljina O. Derix J. Schirrmeister R. T. Schulze-Bonhage A. Auer P. Aertsen A. (2017). Neurolinguistic and machine-learning perspectives on direct speech BCIs for restoration of naturalistic communication. Brain Comput. Interfaces 4, 186–199. 10.1080/2326263X.2017.1330611

[B44] Indefrey P. Levelt W. (2004). The spatial and temporal signatures of word production components. Cognition 92, 101–144. 10.1016/j.cognition.2002.06.001 15037128

[B45] Iqbal S. Khan Y. U. Farooq O. (2015). “EEG based classification of imagined vowel sounds,” in 2015 2nd International Conference on Computing for Sustainable Global Development (INDIACom) (New Delhi), 1591–1594.

[B46] Kellis S. Miller K. Thomson K. Brown R. House P. Greger B. (2010). Decoding spoken words using local field potentials recorded from the cortical surface. J. Neural Eng. 7:056007. 10.1088/1741-2560/7/5/056007 20811093 PMC2970568

[B47] Kiernan M. C. Vucic S. Cheah B. C. Turner M. R. Eisen A. Hardiman O.. (2011). Amyotrophic lateral sclerosis. Lancet 377, 942–955. 10.1016/S0140-6736(10)61156-7 21296405

[B48] Klados M. A. Papadelis C. Lithari C. D. Bamidis P. D. (2009). “The removal of ocular artifacts from EEG signals: a comparison of performances for different methods,” in 4th European Conference of the International Federation for Medical and Biological Engineering, eds Vander Sloten J. Verdonck P. Nyssen M. Haueisen J. (Berlin; Heidelberg: Springer Berlin Heidelberg), 1259–1263.

[B49] Kohnen R. F. Lavrijsen J. C. M. Bor J. H. J. Koopmans R. T. C. M. (2013). The prevalence and characteristics of patients with classic locked-in syndrome in dutch nursing homes. J. Neurol. 260, 1527–1534. 10.1007/s00415-012-6821-y 23306659

[B50] Krizhevsky A. Sutskever I. Hinton G. E. (2017). ImageNet classification with deep convolutional neural networks. Commun. ACM 60, 84–90. 10.1145/3065386

[B51] Leuthardt E. C. Gaona C. M. Sharma M. Szrama N. P. Roland J. L. Freudenberg Z. (2011). Using the electrocorticographic speech network to control a brain-computer interface in humans. J. Neural Eng. 83:036004 10.1088/1741-2560/8/3/036004 PMC3701859 21471638

[B52] Levelt W. J. (1999). Models of word production. Trends Cogn. Sci. 3, 223–232. 10.1016/S1364-6613(99)01319-4 10354575

[B53] Lilly J. M. Olhede S. C. (2009). Higher-order properties of analytic wavelets. IEEE Trans. Signal Process. 57, 146–160. 10.1109/TSP.2008.2007607

[B54] Livezey J. A. Bouchard K. E. Chang E. F. (2019). Deep learning as a tool for neural data analysis: speech classification and cross-frequency coupling in human sensorimotor cortex. PLoS Comput. Biol. 15:e1007091. 10.1371/journal.pcbi.1007091 31525179 PMC6762206

[B55] Martin S. F. Brunner P. Iturrate I. Millán J. Schalk G. Knight R. T.. (2016). Corrigendum: word pair classification during imagined speech using direct brain recordings. Sci. Rep. 7:44509. 10.1038/srep44509 28332505 PMC5362949

[B56] Memarian N. Ferrari P. Macdonald M. J. Cheyne D. Nil L. F. D. Pang E. W. (2012). Cortical activity during speech and non-speech oromotor tasks: a magnetoencephalography (MEG) study. Neurosci. Lett. 527, 34–39. 10.1016/j.neulet.2012.08.030 22926020 PMC4884082

[B57] Moses D. Leonard M. Makin J. Chang E. (2019). Real-time decoding of question-and-answer speech dialogue using human cortical activity. Nat. Commun. 10:3096. 10.1038/s41467-019-10994-4 31363096 PMC6667454

[B58] Nguyen C. H. Karavas G. K. Artemiadis P. (2017). Inferring imagined speech using EEG signals: a new approach using Riemannian manifold features. J. Neural Eng. 15:016002. 10.1088/1741-2552/aa8235 28745299

[B59] Oppenheim G. M. Dell G. S. (2008). Inner speech slips exhibit lexical bias, but not the phonemic similarity effect. Cognition 106, 528–537. 10.1016/j.cognition.2007.02.006 17407776 PMC2435259

[B60] Palmer E. D. Rosen H. J. Ojemann J. G. Buckner R. L. Kelley W. M. Petersen S. E. (2001). An event-related fMRI study of overt and covert word stem completion. NeuroImage 14, 182–193. 10.1006/nimg.2001.0779 11525327

[B61] Porbadnigk A. Wester M. Calliess J. Schultz T. (2009). “EEG-based speech recognition - impact of temporal effects,” in Biosignals eds Encarnação P. Veloso A. (Porto: SciTePress), 376–381.

[B62] Ramsey N. Salari E. Aarnoutse E. Vansteensel M. Bleichner M. Freudenburg Z. (2018). Decoding spoken phonemes from sensorimotor cortex with high-density ECoG grids. NeuroImage 180, 301–311. 10.1016/j.neuroimage.2017.10.011 28993231 PMC6433278

[B63] Rezazadeh Sereshkeh A. Trott R. Bricout A. Chau T. (2017). EEG classification of covert speech using regularized neural networks. IEEE/ACM Trans. Audio Speech Lang. Process. 25, 2292–2300. 10.1109/TASLP.2017.2758164

[B64] Roberts G. Holmes N. Alexander N. Boto E. Leggett J. Hill R. M.. (2019). Towards OPM-MEG in a virtual reality environment. NeuroImage 199, 408–417. 10.1016/j.neuroimage.2019.06.010 31173906 PMC8276767

[B65] Roy Y. Banville H. Albuquerque I. Gramfort A. Falk T. H. Faubert J. (2019). Deep learning-based electroencephalography analysis: a systematic review. J. Neural Eng. 16:051001. 10.1088/1741-2552/ab260c 31151119

[B66] Russakovsky O. Deng J. Su H. Krause J. Satheesh S. Ma S. (2015). ImageNet large scale visual recognition challenge. Int. J. Comput. Vision (IJCV) 115, 211–252. 10.1007/s11263-015-0816-y

[B67] Safieddine D. Kachenoura A. Albera L. Birot G. Karfoul A. Pasnicu A. (2012). Removal of muscle artifact from EEG data: comparison between stochastic (ICA and CCA) and deterministic (EMD and wavelet-based) approaches. EURASIP J. Appl. Signal Process. 2012:127 10.1186/1687-6180-2012-127

[B68] Shorten C. Khoshgoftaar T. M. (2019). A survey on image data augmentation for deep learning. J. Big Data 6, 1–48. 10.1186/s40537-019-0197-0 PMC8287113 34306963

[B69] Shuster L. I. Lemieux S. K. (2005). An fMRI investigation of covertly and overtly produced mono- and multisyllabic words. Brain Lang. 93, 20–31. 10.1016/j.bandl.2004.07.007 15766765

[B70] Simos P. G. Breier J. I. Zouridakis G. Papanicolaou A. (1998). Identification of language-specific brain activity using magnetoencephalography. J. Clin. Exp. Neuropsychol. 20, 706–722. 10.1076/jcen.20.5.706.1127 10079046

[B71] Smith E. Delargy M. (2005). Locked-in syndrome. BMJ 330, 406–409. 10.1136/bmj.330.7488.40 15718541 PMC549115

[B72] Szegedy C. Ioffe S. Vanhoucke V. (2016). “Inception-v4, Inception-ResNet and the impact of residual connections on learning,” in AAAI (Phoenix, AZ), 4278–4284.

[B73] Tadel F. Baillet S. Mosher J. C. Pantazis D. Leahy R. M. (2011). Brainstorm: a user-friendly application for MEG/EEG analysis. Intell. Neurosci. 2011:8797. 10.1155/2011/879716 21584256 PMC3090754

[B74] Vialatte F.-B. Solé-Casals J. Cichocki A. (2008). EEG windowed statistical wavelet scoring for evaluation and discrimination of muscular artifacts. Physiol. Meas. 29, 1435–1452. 10.1088/0967-3334/29/12/007 19001689

[B75] Wang J. Kim M. Hernandez-Mulero A. W. Heitzman D. Ferrari P. (2017). “Towards decoding speech production from single-trial magnetoencephalography (MEG) signals,” in 2017 IEEE International Conference on Acoustics, Speech and Signal Processing (ICASSP) (New Orleans, LA), 3036–3040.

[B76] Wang Y. Wang P. Yu Y. (2018). Decoding english alphabet letters using EEG phase information. Front. Neurosci. 12:62. 10.3389/fnins.2018.00062 29467615 PMC5808334

[B77] Wu S. Zhong S. Liu Y. (2018). Deep residual learning for image steganalysis. Multimedia Tools Appl. 77, 10437–10453. 10.1007/s11042-017-4440-4

[B78] Yoshimura N. Nishimoto A. Belkacem A. N. Shin D. Kambara H. Hanakawa T.. (2016). Decoding of covert vowel articulation using electroencephalography cortical currents. Front. Neurosci. 10:175. 10.3389/fnins.2016.00175 27199638 PMC4853397

[B79] Zetter R. Iivanainen J. Parkkonen L. (2019). Optical co-registration of MRI and on-scalp MEG. Sci. Rep. 9:5490. 10.1038/s41598-019-41763-4 30940844 PMC6445124

[B80] Zhao S. Rudzicz F. (2015). “Classifying phonological categories in imagined and articulated speech,” in 2015 IEEE International Conference on Acoustics, Speech and Signal Processing (ICASSP) (Brisbane, QLD), 992–996.

